# Jianpi Yiqi Busui prescription alleviates myasthenia gravis by regulating Th17 through the TAK1/P38 MAPK/eIF-4E signaling pathway

**DOI:** 10.17305/bb.2025.11546

**Published:** 2025-02-07

**Authors:** Zhuming Chen, Jing Lu, Tianying Chang, Dongmei Zhang, Yibin Zhang, Miao Liu, Tong Wu, Peng Xv, Jian Wang

**Affiliations:** 1 College of Traditional Chinese Medicine, Changchun University of Chinese Medicine, Changchun, China; 2 Department of Neurology, The Affiliated Hospital to Changchun University of Chinese Medicine, Changchun, China; 3 Research Center of Traditional Chinese Medicine, The Affiliated Hospital to Changchun University of Chinese Medicine, Changchun, China; 4 Evidence-Based Office, The Affiliated Hospital to Changchun University of Chinese Medicine, Changchun, China

**Keywords:** Myasthenia gravis, MG, traditional Chinese medicine, TCM, Jianpi Yiqi Busui prescription, JYBP, P38 mitogen-activated protein kinase, P38 MAPK, eukaryotic initiation factor 4E, eIF-4E, Th17 cells, IL-17, CD4^+^ T cells

## Abstract

Jianpi Yiqi Busui Prescription (JYBP), a traditional Chinese medicine formula (TCM), is used in the treatment of myasthenia gravis (MG). However, its mechanisms of action still require further clarification. In this study, an experimental autoimmune MG (EAMG) rat model was established for research. Changes in body weight, forelimb grip strength, Lennon clinical score, and antifatigue ability of EAMG model rats were recorded to evaluate the effectiveness of JYBP. Flow cytometry was utilized to count Th17, Th1, Th2, and Treg cells in lymphocytes. ELISA and RT-qPCR were used to measure acetylcholine receptor antibody (AChR-Ab) and Th17-related cytokines, including IL-17, IL-21, IL-23, TNF-α, TGF-β, IL-1β, and IL-6. Western blot and immunofluorescence staining were used to detect the expression levels of key proteins and their phosphorylated forms, such as transforming growth factor beta-activated kinase 1 (TAK1), P38 mitogen-activated protein kinase (P38 MAPK), and eukaryotic initiation factor 4E (eIF-4E). The results indicate that JYBP can increase the body weight of EAMG model rats, improve grip strength and antifatigue ability, and reduce the Lennon clinical score and AChR-Ab concentration. Mechanistic studies indicate that JYBP can inhibit the differentiation of CD4^+^ T cells into Th17 and Th1, promote their differentiation into Th2 and Treg, and regulate the expression of Th17-related cytokines. Further research shows that JYBP can reduce the expression of related proteins in the TAK1/P38 MAPK/eIF-4E signaling pathway. In conclusion, JYBP can alleviate the condition of EAMG model rats, positively affecting MG treatment. The inhibitory effect of JYBP on the differentiation of CD4^+^ T cells into Th17 may be related to the TAK1/P38 MAPK/eIF-4E signaling pathway.

## Introduction

Myasthenia gravis (MG) is an autoimmune disease characterized by neuromuscular junction (NMJ) transmission disorders mediated by T cells, dependent on B cells, and involving cytokines and complement [1]. Acetylcholine receptor (AChR) antibodies are considered the most common pathogenic autoantibodies. However, other autoantibodies targeting components of the postsynaptic membrane—such as muscle-specific receptor tyrosine kinase (MuSK), low-density lipoprotein receptor-related protein 4 (LRP4), and ryanodine receptor (RyR)—have also been implicated in the pathogenesis of MG. These autoantibodies can interfere with AChR clustering, impair AChR function, and disrupt NMJ signaling [2, 3].

This study will explore MG with AChR antibody positivity using the experimental autoimmune MG (EAMG) rat model. The primary symptom of MG is muscle weakness in the affected areas, commonly presenting as drooping eyelids, double vision, difficulty chewing and swallowing, and limb weakness. In severe cases, dyspnea may occur. These symptoms typically follow a pattern of being milder in the morning and worsening throughout the day. Based on symptom presentation, MG can be classified as either ocular MG (OMG) or generalized MG (GMG) [4].

The incidence of MG ranges from 10 to 29 cases per million, with a prevalence of 100–350 cases per million. It is more common in women than in men [5]. Due to its chronic nature, tendency to relapse, and challenges in treatment, MG remains a difficult disease to manage globally, causing significant patient suffering and imposing a substantial economic burden on both families and society [6, 7].

Current treatment options for MG primarily focus on medication and surgery. Medical therapies include acetylcholinesterase inhibitors, glucocorticoids, non-steroidal immunosuppressants, and targeted biologics. Surgical treatments mainly involve thymectomy, with autologous hematopoietic stem cell transplantation also considered in certain cases. In severe, life-threatening situations, intravenous immunoglobulin (IVIG) and plasma exchange therapy may be recommended [8].

However, these treatments come with notable limitations, such as significant side effects from long-term medication use, susceptibility to recurrent episodes, unclear long-term risks associated with surgical interventions, and high treatment costs [9–12]. Furthermore, standard therapies may not always achieve sufficient symptom control or remission, even with appropriate dosing and treatment duration, posing ongoing challenges for both patients and healthcare providers. This underscores the need to explore innovative and alternative immunomodulatory therapies for managing more complex cases of MG [13].

In China, traditional Chinese medicine (TCM) is frequently used to treat MG, offering advantages, such as good efficacy, minimal side effects, lower treatment costs, and a reduced likelihood of recurrence [14]. One such TCM approach is the Jianpi Yiqi Busui Prescription (JYBP), also known as Qi-Shen-Di-Huang, a hospital preparation from the Affiliated Hospital of Changchun University of Chinese Medicine. JYBP is composed of various herbs, including Astragalus and Codonopsis. Some studies suggest that active ingredients in JYBP, such as Astragaloside IV, may alleviate MG symptoms by regulating mitochondrial function and gut microbiota [15, 16].

However, the precise mechanisms by which JYBP, as a complete TCM compound, exerts therapeutic effects on MG remain unclear. Therefore, modern biomedical techniques are needed to investigate the potential mechanisms of JYBP, with the aim of enhancing its therapeutic efficacy in MG treatment.

Studies have shown that changes in Th1, Th2, Th17, and Treg cells are important factors influencing the development of MG [17], all of which differentiate from CD4^+^ T cells [18]. Inhibiting the excessive differentiation of Th17 cells can effectively alleviate MG symptoms [19]. Some studies suggest that MG can also be mitigated by regulating cytokines, such as TNF-α, IL-1β, IL-6, and TGF-β [20, 21]. These cytokines influence the activation of the p38 mitogen-activated protein kinase (p38 MAPK) signaling pathway via transforming growth factor beta-activated kinase 1 (TAK1) and are closely associated with Th17 differentiation [22, 23]. In this study, JYBP is shown to have a regulatory effect on these cytokines.

Additionally, research indicates that during translation, p38 MAPK can promote IL-17 expression and Th17 cell differentiation by activating eukaryotic initiation factor 4E (eIF-4E) in CD4^+^ T cells [24]. Our network pharmacology research further suggests that MAPK is a key signaling pathway through which JYBP exerts its therapeutic effects on MG [25, 26].

Despite these findings underscoring the role of the p38 MAPK signaling pathway in MG pathogenesis, there is still limited research on how JYBP modulates this pathway to influence the differentiation of CD4^+^ T cells into Th17 cells. Based on these results and prior studies, this research proposes that JYBP’s therapeutic mechanism in MG may involve inhibiting the differentiation of CD4^+^ T cells into Th17 cells via the TAK1/p38 MAPK/eIF-4E signaling pathway. Investigating this hypothesis is expected to address this research gap and offer new insights and potential treatment strategies for MG.

## Materials and methods

### Establishment of the EAMG rat model

The EAMG rat model was established using an active immunization method. Sixty SPF female Lewis rats, aged 6–8 weeks and weighing 160–180 g, were selected (Beijing Charles River Laboratory Animal Technology Co., Ltd., license number: SCXK 2016-0006; qualification certificate number: No. 110011210109257156). The research was conducted in the SPF barrier environment of the Experimental Animal Center at Changchun University of Chinese Medicine. The rats were housed in cages (five per cage) under a 12-h light/dark cycle with the following environmental conditions: temperature of 20–26 ^∘^C, daily temperature fluctuation ≤4 ^∘^C, relative humidity of 40%–70%, ventilation frequency of 20 times per hour, air velocity at cage level ≤0.2 m/s, minimum static pressure difference ≥10 Pa, air cleanliness at class seven, ammonia concentration ≤14 mg/m^3^, noise ≤60 dB, working illumination at 150 Lx, and animal illumination at 15–20 Lx. Throughout the study, the rats had ad libitum access to food and water.

**Figure 1.**
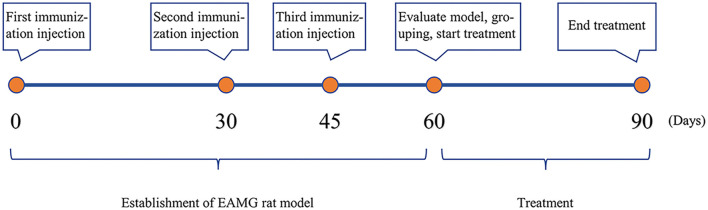
**Key time points for the establishment and treatment of the EAMG rat model.** Rats accepted immunization injections on days 0, 30, and 45, and model evaluation and grouping were performed on day 60. Subsequently, treatment was carried out for 30 days. EAMG: Experimental autoimmune myasthenia gravis.

Using the random number table method, eight rats were randomly selected for the adjuvant control group, while the remaining 52 rats were injected with antigen emulsion to induce the model. A sterile syringe was used to subcutaneously inject 40 µL of immune antigen emulsion at five different sites: the hind footpads, tail base, back, and nape of the neck, for a total volume of 200 µL per rat. Each 200 µL of antigen emulsion was prepared by thoroughly mixing 100 µL of complete Freund’s adjuvant (CFA, Sigma-Aldrich, USA; Cat: F5881), 100 µL of phosphate-buffered saline (PBS, Cytiva, USA; Cat: AH30026713), 50 µg of AChRα97-116 peptide (Shanghai Qiangyao Biotechnology Co., Ltd., Cat: 04010001391), and 1 mg of Mycobacterium tuberculosis H37Ra dry powder (BD Difco, USA; Cat: 231141-6). The emulsion used for the control group did not contain AChRα97-116 peptide or H37Ra dry powder. Following the initial immunization injection, the second and third injections were administered on days 30 and 45, respectively, with model evaluation conducted on day 60 (Figure 1).

**Table 1:** List of JYBP components

**Latin name**	**Chinese name**	**Family**	**Medicinal part**
*Astragalus membranaceus* (Fisch.) Bge.	Huang qi	Leguminosae	Root
*Codonopsis pilosula* (Franch.) Nannf.	Dang shen	Campanulaceae	Root
*Pheretima aspergillum* (E. Perrir)	Di long	Megascolecidae	Whole body
*Atractylodes macrocephala* Koidz.	Bai zhu	Asteraceae	Root
*Angelica sinensis* (Oliv.) Diels.	Dang gui	Umbelliferae	Root
*Rehmannia glutinosa* Libosch	Shen di	Scrophulariaceae	Root
*Lycium barbarun* L.	Gou qizi	Solanaceae	Fruit
*Dioscorea opposite* Thunb.	Shan yao	Dioscoreaceae	Root
*Cimicifuga heracleifolia* Kom.	Sheng ma	Ranunculaceae	Root
*Bupleurum chinense* DC.	Chai hu	Umbelliferae	Root
*Glycyrrhiza uralensis* Fisch.	Zhi ganchao	Leguminosae	Root

JYBP: Jianpi Yiqi Busui Prescription.

### Evaluation of the EAMG rat model

The EAMG rat model was considered successfully established if the following five criteria were met: ① weight loss; ② a Lennon clinical score of at least one point [27]; ③ a significant decrease in forelimb grip strength; ④ a reduction of more than 10% in low-frequency repetitive nerve stimulation (RNS) values; and ⑤ a significant increase in peripheral blood serum AChR antibody (AChR-Ab) concentration. All data had to show statistically significant differences compared to the control group. Forelimb grip strength was assessed using a grip strength meter (Yiyan Technology Development Co., Ltd., Model: YLS-13A). Each rat was tested five times, and the average value was used for statistical analysis. RNS testing was performed on the gastrocnemius muscle of both groups using an electromyography-evoked potential instrument (Oxford Instruments, Model: NHK30). To ensure objective and reliable results, two pre-trained technicians, who were not involved in the experiment, independently conducted these measurements.

After the model evaluation was completed, the qualified model rats were randomly divided into a model group and JYBP low-, medium-, and high-dose groups using a random number table. Additionally, since prednisone is one of the primary drugs currently used to treat MG and has a regulatory effect on the P38 MAPK signaling pathway [28, 29], a prednisone group was established to better evaluate the effectiveness of JYBP in treating MG.

### Interventions for EAMG model rats

JYBP is composed of various medicinal herbs and animal-derived ingredients. Detailed information on the prescription is provided in Table 1. JYBP is processed and produced by Yatai Yongantang Pharmaceutical Co., Ltd. in Jilin Province (Lot: 210303T). The gradient dosing of JYBP was determined using the equivalent dose conversion formula:

Animal dose ═ Human dose × (Human Km factor/Animal Km factor).

In this formula, the human Km factor is 37, and the rat Km factor is six. Based on this calculation, the equivalent dose for rats is approximately 6.2 times higher than the dose for humans. The standard dose of JYBP for treating MG patients is 1.55 g/kg/day, making the equivalent dose for rats approximately 9.6 g/kg/day [30]. This dose was used for the medium-dose group, while the low-dose group received half of this amount, and the high-dose group received double. The final drug groupings and dosages for treating EAMG model rats were as follows: Prednisone group: 5.4 mg/kg/day JYBP low-dose group: 4.8 g/kg/day JYBP medium-dose group: 9.6 g/kg/day JYBP high-dose group: 19.2 g/kg/day Physiological saline (10 mL/kg/day) was used as a solvent to dissolve the drugs for gavage administration. Both the model group and the control group received only physiological saline (10 mL/kg/day). These interventions began after model evaluation was completed, and all groups underwent gavage treatment for 30 days (Figure 1).

### Evaluation of treatment efficacy

The efficacy of JYBP in treating EAMG model rats was evaluated based on body weight, forelimb grip strength, rotarod performance, Lennon clinical score, and AChR-Ab concentration. Body weight was measured every three days, the Lennon clinical score was assessed every seven days, and the remaining parameters were tested once at the end of the treatment. A rodent rotarod test instrument (Yiyan Technology Development Co., Ltd., Model: YLS-4D) was used to assess fatigue resistance in each group of rats, with results calculated based on the time each rat remained on the rotating rod. All measurements were conducted by two pre-trained technicians who were not involved in the study, following the same protocol used in the model evaluation.

### Flow cytometry

Flow cytometry was used to detect the numbers of Th17 (CD4^+^IL−17+ T), Th1 (CD4^+^IFN−γ+ T), Th2 (CD4^+^IL−4+ T), and Treg (CD4^+^CD25+Foxp3+ T) cells. Lymphocytes were extracted from the spleen tissue of rats using Ficoll (Beijing Solable Science and Technology Co., Ltd., Cat: P8610). For each sample, a CD4 antibody and 50 µL of staining buffer were added (for Treg detection, a CD25 antibody was also included). The samples were incubated in the dark for 30 min, followed by the addition of 1 mL of staining buffer and centrifugation. The supernatant was discarded, and the pellet was resuspended in 200 µL of flow buffer. Unstained and CD4 single-stained samples were retained as controls for threshold adjustment during detection. To the remaining samples, 500 µL of fixation/permeabilization diluent was added, followed by incubation in the dark for 50 min. Then, 500 µL of permeabilization buffer was added, and the samples were centrifuged. After discarding the supernatant, 100 µL of permeabilization buffer, along with IL-17, IFN-γ, IL-4, or Foxp3 antibodies, was added. After incubating in the dark for 50 min, 2 mL of permeabilization buffer was added, followed by centrifugation. The supernatant was discarded, and 200 µL of staining buffer was added and mixed thoroughly. Samples were kept in the dark until detection.

A flow cytometer system (Beckman Coulter Biotechnology Co., Ltd., Model: A00-1-1102) was used to determine cell counts. For accurate flow cytometry analysis, quadrant thresholds were established using unstained control samples. Single-stained controls were employed to calibrate the compensation matrix, with each fluorophore’s single-stained sample used to correct for spectral overlap between channels. After applying compensation, marker thresholds were adjusted to ensure precise classification of cell populations. These threshold settings remained consistent across all experimental groups, and quadrant divisions were based on the compensated fluorescence intensities. Final data analysis was conducted using these adjusted thresholds to ensure accurate population gating. Detailed gating strategies and compensation results are provided in the supplementary material. All antibodies were purchased from BioLegend (USA), and the staining kits were obtained from Thermo Fisher Scientific (USA).

### ELISA

Blood samples collected from rats were centrifuged at 3000 rpm for 15 min at 4 ^∘^C to separate the serum. The expression levels of AChR-Ab, IL-17, IL-6, TGF-β, TNF-α, IL-1β, IL-21, and IL-23 in the serum samples were measured using a microplate reader (BioTek Corporation, United States, Model: Box 998) and ELISA kits. The AChR-Ab ELISA kits were purchased from Shanghai Enzyme-linked Biotechnology Co., Ltd., while all other ELISA kits were obtained from Yanko Biological Technology Co., Ltd. Except for AChR-Ab, the other cytokines are closely associated with the differentiation of Th17 cells. IL-17 is primarily secreted by Th17 cells [31]. Changes in the expression levels of TGF-β and IL-6 influence the differentiation of CD4^+^ T cells into Th17 cells, while elevated levels of IL-1β and TNF-α can enhance Th17 differentiation induced by TGF-β and IL-6 [32]. IL-21 and IL-23 are considered key factors in the maintenance and proliferation of Th17 cells [19, 33].

### RT-qPCR

Total RNA was extracted from rat spleen tissue using the TRIZOL method. The RNA concentration from each sample was measured, adjusted accordingly, and reverse-transcribed into cDNA using the FastKing kit (Tiangen Biochemical Technology Co., Ltd., Cat: KR118) with a gradient thermal cycler (Eppendorf Corporation, Germany, Model: 6331). For quantitative analysis, the SYBR Green kit (Tiangen Biochemical Technology Co., Ltd., Cat: FP205) was utilized. According to the kit’s instructions, the PCR reaction mixture for each sample consisted of 1 µL of forward primer, 1 µL of reverse primer (for the respective target genes), 5 µL of SYBR Green mix, 2 µL of RNase-free ddH_2_O, and 1 µL of cDNA. These components were added to the wells of a PCR detection plate. RT-qPCR was performed using a CFX96 Optics Module detection instrument (BIO-RAD Corporation, USA). The target genes analyzed included IL-17, IL-6, TGF-β, TNF-α, IL-1β, IL-21, and IL-23. GAPDH was used as the housekeeping gene for normalization. The relative mRNA expression levels were calculated using the 2^-ΔΔCt^ method. The primer sequences were designed and synthesized by Shanghai Sangon Biotech Co., Ltd., and are listed in Table 2.

**Table 2:** List of Primer sequences used in this study

**Gene**	**Forward primer**	**Reverse primer**
*IL-17*	CTGATGCTGTTGCTGCTACTGAAC	CGGCGTTTGGACACACTGAAC
*IL-21*	CCTCAGCTGTGCCAACAAGTC	TCCTGAACTTCAACAGCTCCACA
*IL-23*	CAGCGTTCTCTTCTCCGT	CGTTGGCACTAAGGGCTC
*TNF-α*	AAGGACACCATGAGCACTGAAAGC	AGGAAGGAGAAGAGGCTGAGGAAC
*TGF-β*	TCAGACATTCGGAAGCAGTG	ATTCCGTCTCCTTGGTTCAGC
*IL-1β*	CACTACAGGCTCCGAGATGAACAAC	TGTCGTTGCTTGGTTCTCCTTGTAC
*IL-6*	GCCAGTTGCCTTCTTGGGAC	TGGTCTGTTGTGGGTGGTATCC
*GAPDH*	TGGTGAAGCAGGCATCTGA	TGCTGTTGAAGTCGCAGGAG

### Western blot

Proteins were extracted from rat spleen tissue using RIPA lysis buffer (Beyotime Biotechnology Co., Ltd., Cat: P0017), and their concentrations were determined and adjusted using a BCA kit (Beyotime Biotechnology Co., Ltd., Cat: P0011). Subsequently, 40 µg of protein was separated on a 12% SDS-PAGE gel and transferred onto PVDF membranes (Thermo Fisher Scientific, USA, Cat: LC2007). The membranes were then blocked with 5% BSA at room temperature for 1 h. Antibody diluent (Beyotime Biotechnology Co., Ltd., Cat: P0256) was used to dilute all antibodies at the following ratios: TAK1 (1:500), p-TAK1 (1:1000), P38 MAPK (1:2000), p-P38 MAPK (1:1000), eIF-4E (1:500), p-eIF-4E (1:1000), and GAPDH (1:5000). The membranes were incubated with the primary antibodies overnight at 4 ^∘^C. The following day, the membranes were incubated with secondary antibodies at room temperature for 1 h. Protein bands were visualized using chemiluminescent imaging software (ProteinSimple Corporation, USA, Model: FluorChem M), and further quantitative analysis was performed using ImageJ. All antibodies used in the Western blot were purchased from Sanyin Biotechnology Co., Ltd.

### Immunofluorescence staining

The rat spleen tissue was embedded in optimal cutting temperature compound, sectioned, and then fixed with 4% paraformaldehyde (Beijing Solarbio Technology Co., Ltd., Cat: P1110). The samples were treated with 0.5% Triton X-100 cell lysis buffer (Thermo Fisher Scientific, Cat: 85112) for 15 min and rinsed with PBS. A 5% BSA solution was used to dilute the primary antibodies at the following ratios: CD4 (1:100), p-TAK1 (1:100), p-P38 (1:100), p-eIF-4E (1:50), and IL-17 (1:50). The samples were then incubated with the primary antibodies overnight at 4 ^∘^C. The following day, the samples were washed with PBS and incubated with the secondary antibodies at room temperature in the dark for 2 h. After incubation, DAPI (Thermo Fisher Scientific, Cat: D1306) was added, and the samples were incubated in the dark at room temperature for 10 min. Finally, the samples were washed with PBS, mounted on slides, and observed under a fluorescence microscope (Thermo Fisher Scientific, Model: M7000). ImageJ was used to quantify the co-localization of the CD4 signal with p-TAK1, p-P38 MAPK, and p-eIF-4E, as well as the overall expression of IL-17. All antibodies used for immunofluorescence staining were purchased from Sanyin Biotechnology Co., Ltd.

### Ethical statement

This study was approved by the Experimental Animal Ethics Committee of Changchun University of Chinese Medicine (Approval No. 2022401).

### Statistical analysis

Data from different groups were analyzed using SPSS Statistics 26.0 (IBM Corporation, Armonk, NY, USA) and GraphPad Prism 9.5 (GraphPad Software, San Diego, CA, USA). Results are presented as mean ± standard deviation. Depending on the homogeneity of variance and the normality of data distribution, comparisons between two groups were performed using either the *t*-test or the Wilcoxon rank-sum test. Comparisons among multiple groups were conducted using one-way ANOVA, followed by Tukey’s post hoc test. For repeated measures data, two-way repeated measures ANOVA was used, also followed by Tukey’s post hoc test. A *P* value < 0.05 was considered statistically significant.

## Results

### Evaluation of the EAMG rat model

**Figure 2.**
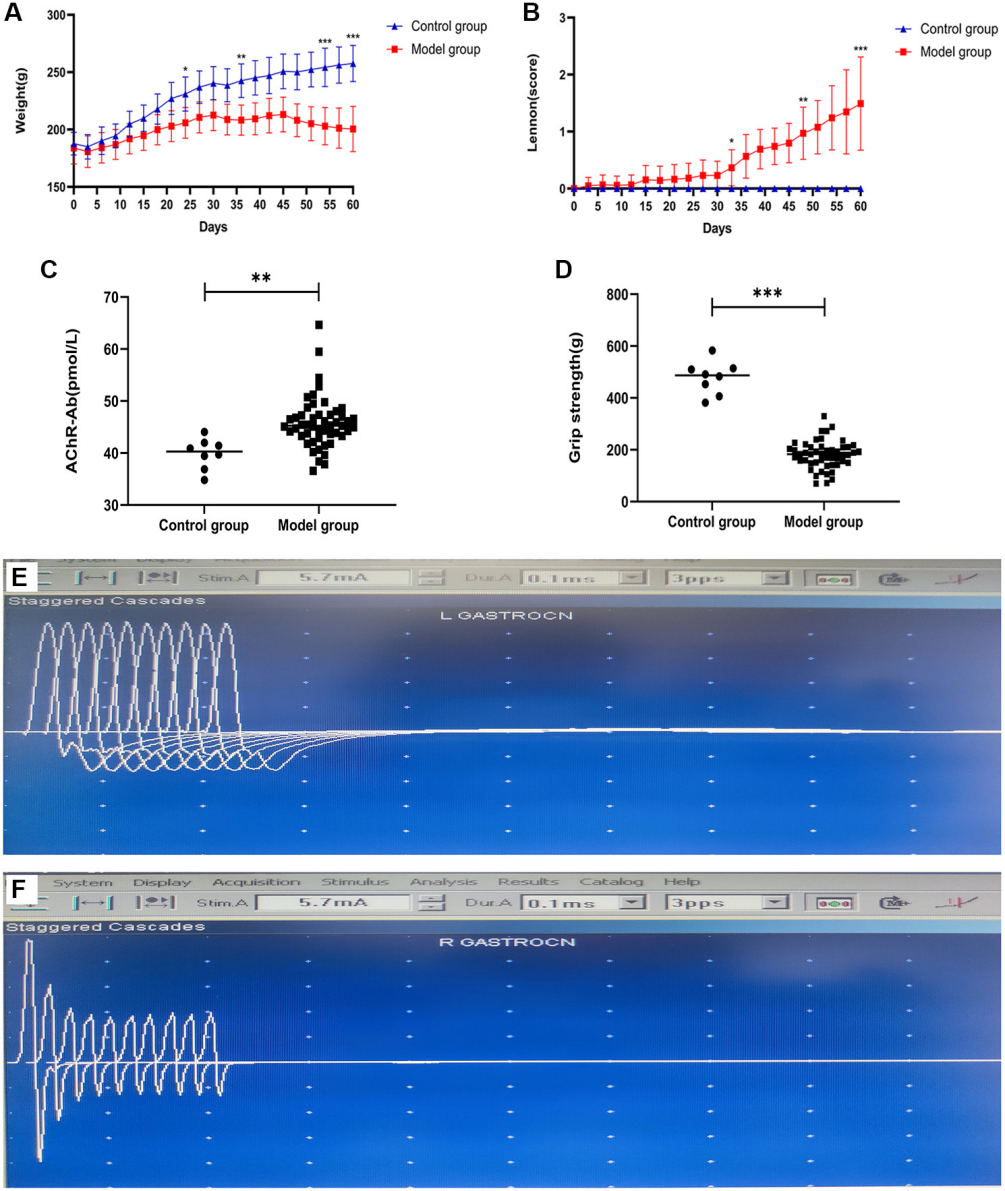
**Evaluation of the EAMG rat model.** (A) Body weight; (B) Lennon clinical score; (C) AChR-Ab concentration was detected by ELISA; (D) Forelimb grip strength; (E) The RNS of the control group without a significant change in action potentials; (F) The RNS of the model group with a reduction exceeding 10% in action potentials. *n* (control group) ═ 8; *n* (model group) ═ 42. *P < 0.01, **P < 0.01, ***P < 0.001. EAMG: Experimental autoimmune myasthenia gravis; RNS: Repetitive nerve stimulation; AchR-Ab: Acetylcholine receptor antibody.

As the number of antigen emulsion injections increased, symptoms related to MG in the model group rats progressively worsened. These symptoms included arching of the back, weakness in raising the neck, limp and weak limbs, difficulty gripping and walking, emaciation, body tremors, pale skin and mucous membranes, disheveled and dry hair, abnormal tooth growth due to reduced food intake, and even rapid breathing. Compared to the control group, the model group rats exhibited significant fluctuations and decreases in body weight following the second and third immunization injections (Figure 2A).

The Lennon clinical scores for the model group showed a marked increase, corresponding with the progressive worsening of disease symptoms and a decline in forelimb grip strength (Figure 2B). To evaluate the model, an AChR-Ab ELISA kit was used to measure AChR-Ab concentrations in rat serum, revealing significantly higher levels in the model group compared to the control group (Figure 2C).

Forelimb grip strength and repetitive RNS were also assessed to enhance the objectivity of the model evaluation. Results indicated that the forelimb grip strength of the model group rats was significantly weaker than that of the control group (Figure 2D). Furthermore, while the control group showed no significant changes in RNS action potentials (Figure 2E), the model group exhibited a gradual decay, with reductions exceeding 10% (Figure 2F).

Out of all the rats in the model group, two died due to severe illness, and another eight were excluded during model evaluation for not meeting the criteria. Ultimately, 42 rats were successfully modeled. These rats were randomly assigned to the following groups: prednisone group (*n* ═ 8), JYBP low-dose group (*n* ═ 8), JYBP medium-dose group (*n* ═ 8), JYBP high-dose group (*n* ═ 8), and model group (*n* ═ 10).

### The efficacy of JYBP in treating the EAMG model rats

**Figure 3.**
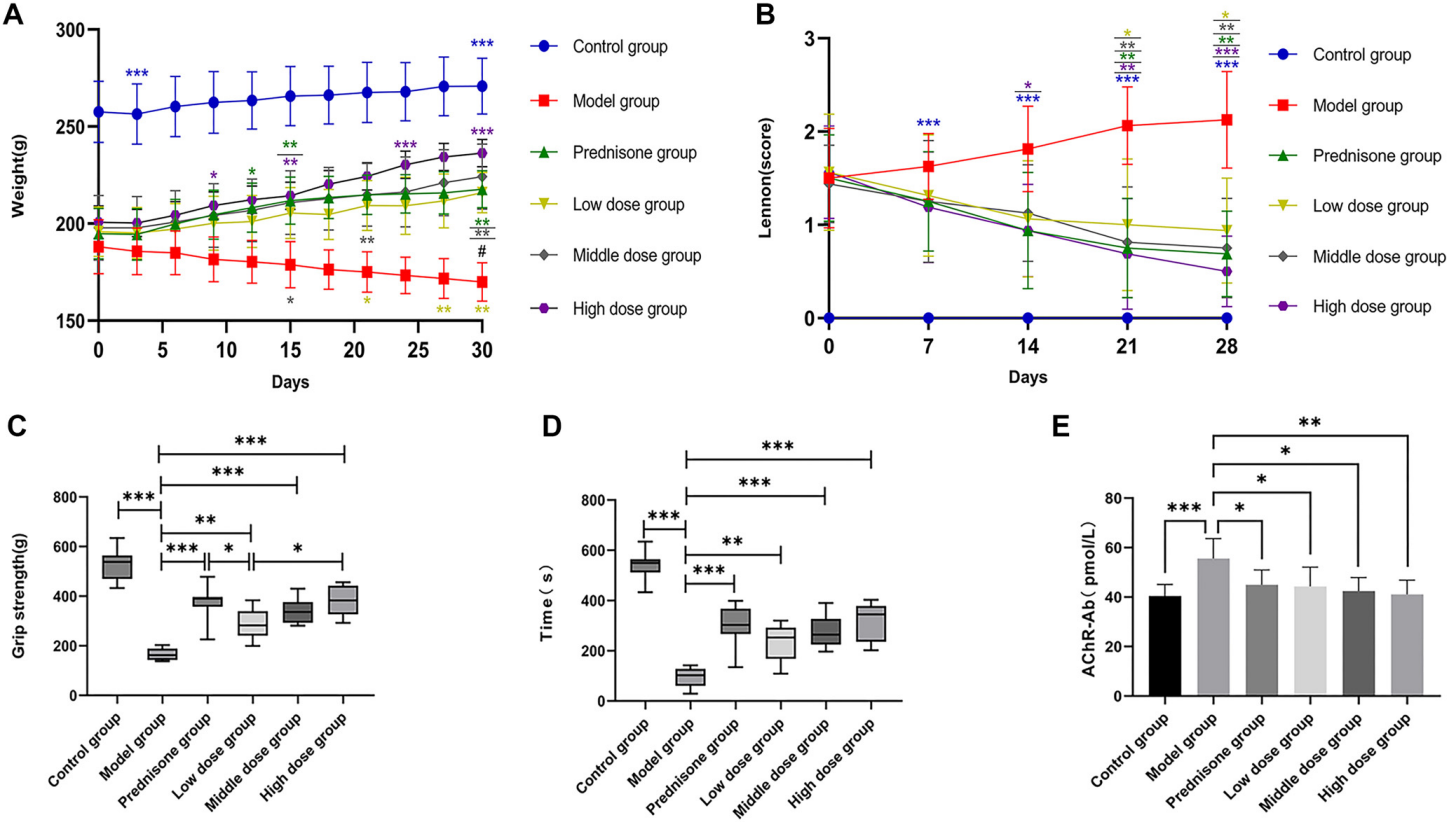
**The efficacy of JYBP in treating the EAMG model rats.** (A) Body weight; (B) Lennon clinical score; (C) Forelimb grip strength; (D) Fatigue resistance was observed by rotarod test; (E) AChR-Ab concentration was detected by ELISA. *n* ═ 8. *P < 0.05, **P < 0.01, ***P < 0.001 compared to the model group. The color of the *P represents different groups. ^#^*P* < 0.05 (Comparison between the low-dose group and the high-dose group). JYBP: Jianpi Yiqi Busui Prescription; EAMG: Experimental autoimmune myasthenia gravis; AchR-Ab: Acetylcholine receptor antibody.

After the treatment began, the body weight of the rats in each drug intervention group gradually increased compared to the model group, with the JYBP high-dose group showing the most significant increase. Notably, the prednisone group experienced faster weight gain during the first two weeks of treatment, but this gain slowed considerably in the later stages (Figure 3A). In contrast to the body weight trends, the Lennon clinical scores of the rats in each drug intervention group decreased after treatment initiation, indicating a gradual recovery of muscle strength (Figure 3B). By the end of the treatment, the forelimb grip strength of rats in the drug intervention groups was higher than that in the model group, with the prednisone and high-dose groups demonstrating the most significant improvements (Figure 3C). Rotarod test analysis further revealed that the duration of stay on the rod was longer for rats in all drug intervention groups compared to the model group (Figure 3D). Additionally, post-treatment serum analysis showed that AChR-Ab concentrations in all drug intervention groups were lower than those in the model group (Figure 3E).

### The influences of JYBP on Th17, Th1, Th2, Treg

**Figure 4.**
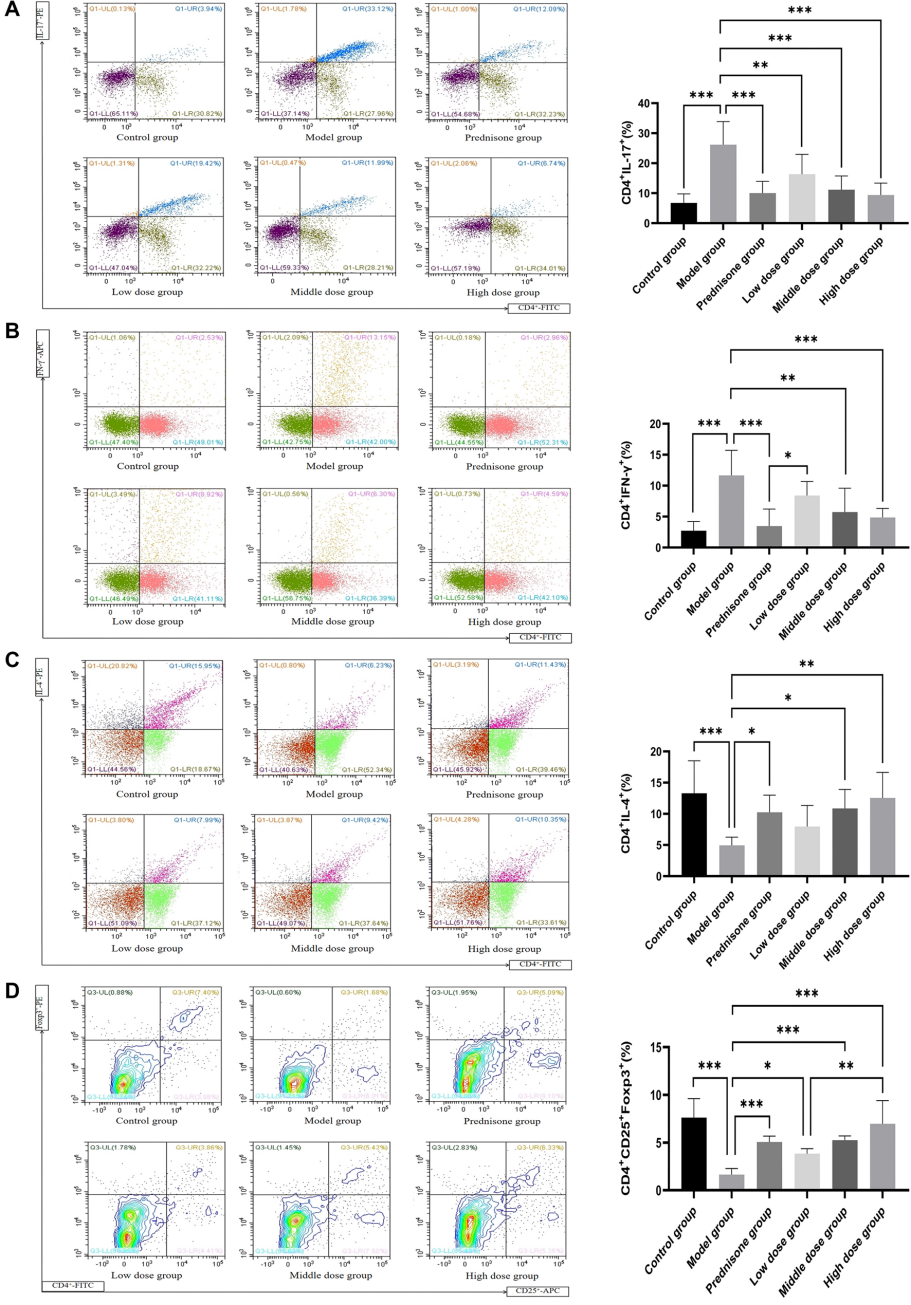
**The influence of JYBP on Th17, Th1, Th2, and Treg cells.** (A) Th17; (B) Th1; (C) Th2; (D) Treg. The flow cytometry plots are shown on the left, with corresponding statistical analysis results are presented on the right. *n* ═ 8. *P < 0.05, **P < 0.01, ***P < 0.001. JYBP: Jianpi Yiqi Busui Prescription.

The flow cytometry results for Th17, Th1, Th2, and Treg cells in rat splenic lymphocytes, along with the corresponding statistical analyses, are presented in Figure 4. Compared to the control group (Th17: 6.77 ± 3.00; Th1: 2.72 ± 1.49; Th2: 13.30 ± 5.20; Treg: 7.61 ± 2.00), rats in the model group showed significant increases in Th17 and Th1 cells by 287% (26.16 ± 7.72) and 329% (11.66 ± 4.06), respectively. Conversely, Th2 and Treg cells decreased by 63% (4.94 ± 1.32) and 78% (1.65 ± 0.64), respectively. Following treatment, compared to the model group, the prednisone group and the JYBP low-, medium-, and high-dose groups exhibited reductions in Th17 cells by 62% (10.01 ± 3.96), 37% (16.35 ± 6.58), 57% (11.18 ± 4.54), and 64% (9.37 ± 4.01), respectively; reductions in Th1 cells by 70% (3.46 ± 2.77), 28% (8.40 ± 2.28), 51% (5.72 ± 3.86), and 58% (4.87 ± 1.46), respectively; increases in Th2 cells by 107% (10.25 ± 2.75), 61% (7.96 ± 3.38), 120% (10.86 ± 3.05), and 154% (12.56 ± 4.09), respectively; and increases in Treg cells by 207% (5.06 ± 0.63), 141% (3.97 ± 0.76), 219% (5.26 ± 0.44), and 323% (6.97 ± 2.42), respectively. Overall, the regulatory effects on these cell populations were more pronounced in the prednisone, JYBP medium-dose, and JYBP high-dose groups.

### The influences of JYBP on Th17-related cytokines

**Figure 5.**
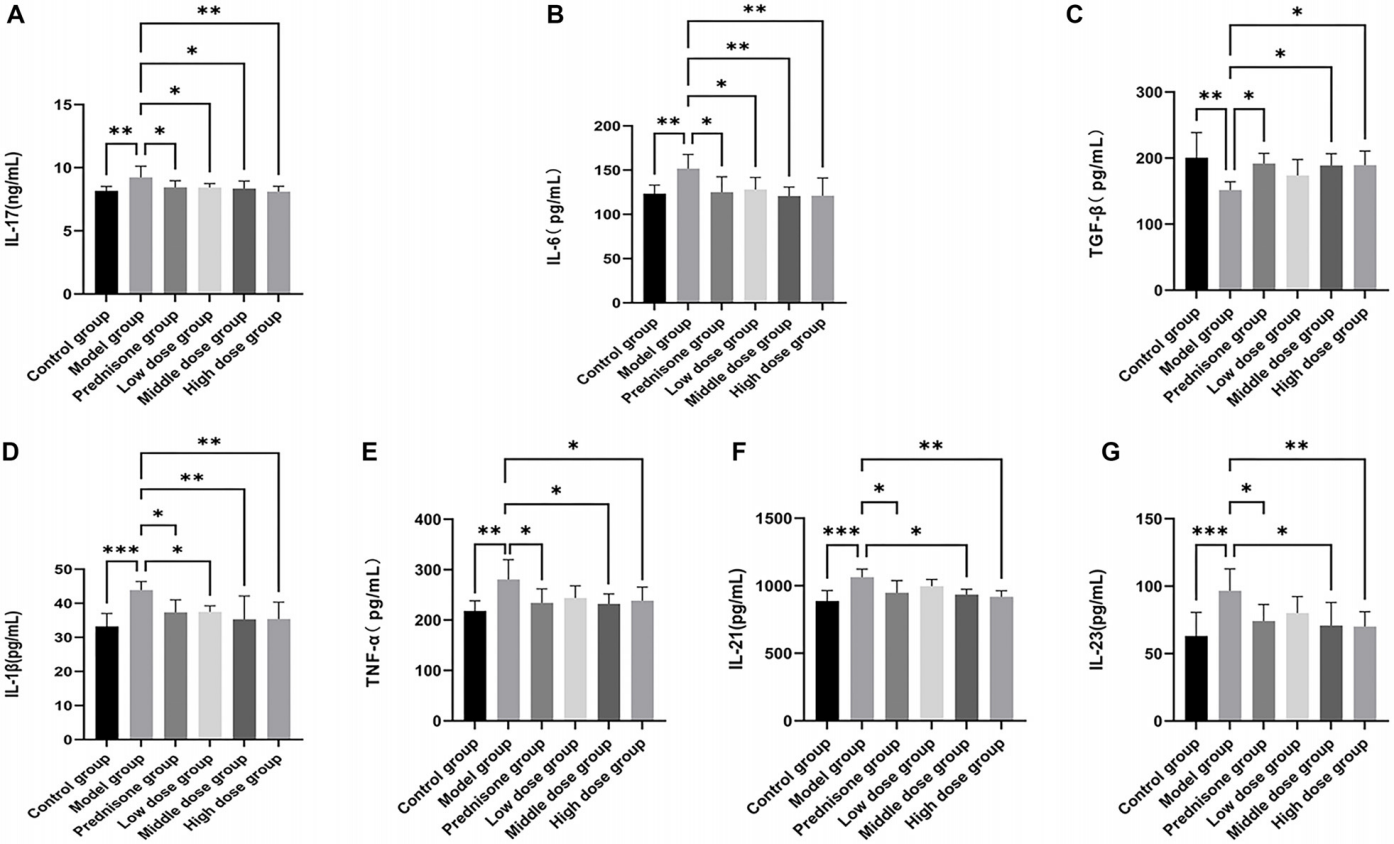
**The effect of JYBP on the protein expression of Th17-related cytokines.** (A) IL-17; (B) IL-6; (C) TGF-β; (D) IL-1β; (E) TNF-α; (F) IL-21; (G) IL-23. They were measured using ELISA. *n* ═ 8. *P < 0.05, **P < 0.01, ***P < 0.001. JYBP: Jianpi Yiqi Busui Prescription.

The ELISA results for Th17-related cytokines in each group are presented in Figure 5. The levels of IL-17, IL-6, IL-1β, TNF-α, IL-21, and IL-23 were elevated in the model group rats compared to the control group, while drug intervention led to a reduction in these cytokine levels. Conversely, TGF-β levels were decreased in the model group relative to the control group but increased following drug intervention. It is worth noting that although TNF-α, TGF-β, IL-21, and IL-23 levels also fluctuated in the low-dose JYBP group, these changes were not statistically significant compared to the control group (*P* > 0.05).

**Figure 6.**
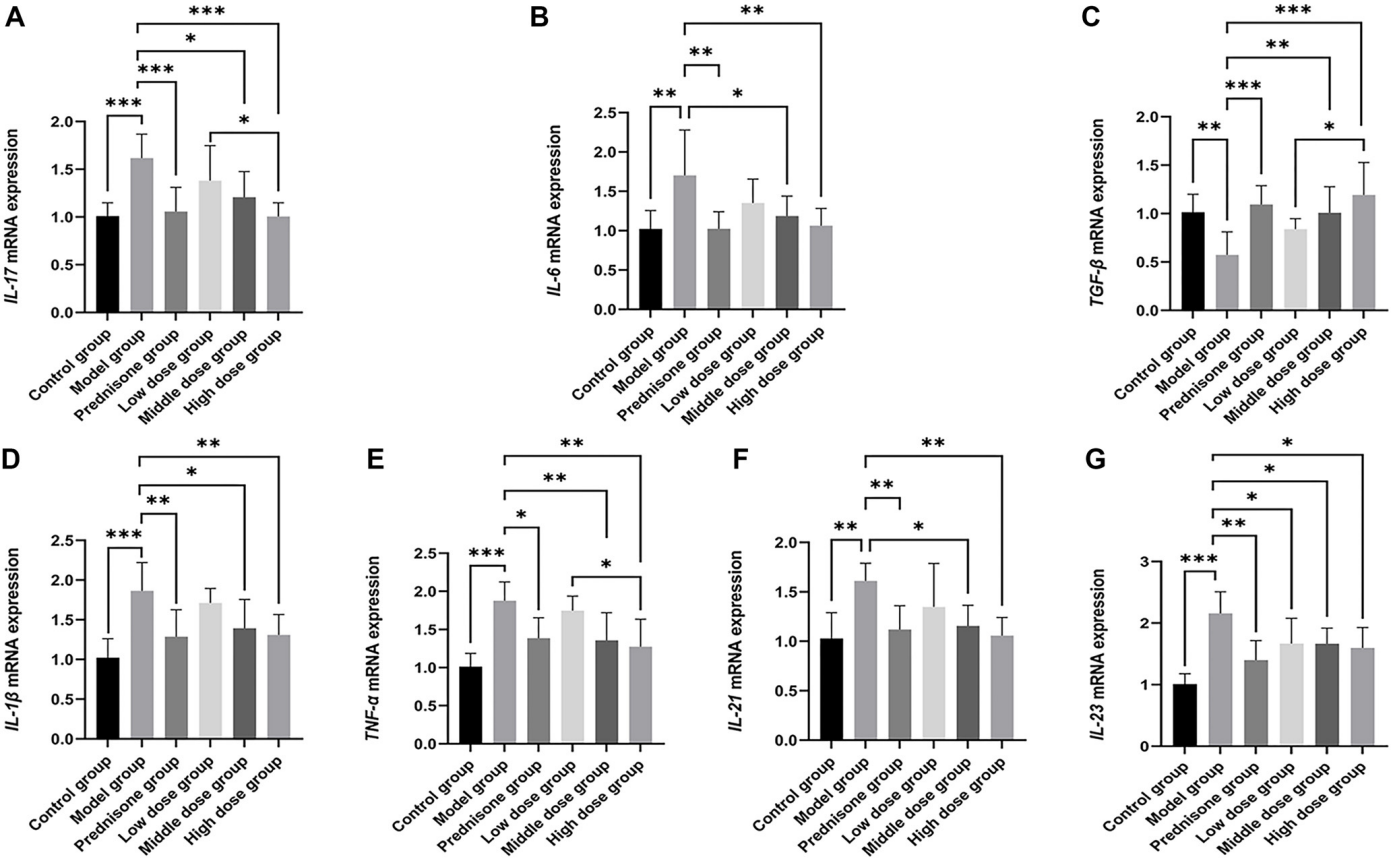
**The effect of JYBP on the mRNA expression of Th17-related cytokines.** (A) *IL-17* mRNA; (B) *IL-6* mRNA; (C) *TGF-β* mRNA; (D) *IL-1β* mRNA; (E) *TNF-α* mRNA; (F) *IL-21* mRNA; (G) *IL-23* mRNA. They were measured using RT-qPCR. *n* ═ 8. *P < 0.05, **P < 0.01, ***P < 0.001. JYBP: Jianpi Yiqi Busui Prescription.

The mRNA levels of Th17-related cytokines in each group, as detected by RT-qPCR, are shown in Figure 6. The trends observed are consistent with the results obtained from ELISA. However, an interesting phenomenon warrants attention: while the mRNA levels of IL-17, IL-6, and IL-1β in the JYBP low-dose group did not show statistically significant differences compared to the model group (*P* > 0.05), the ELISA results for these cytokines demonstrated statistically significant differences (*P* < 0.05).

### JYBP regulates Th17 through the TAK1/P38 MAPK/eIF-4E signaling pathway

**Figure 7.**
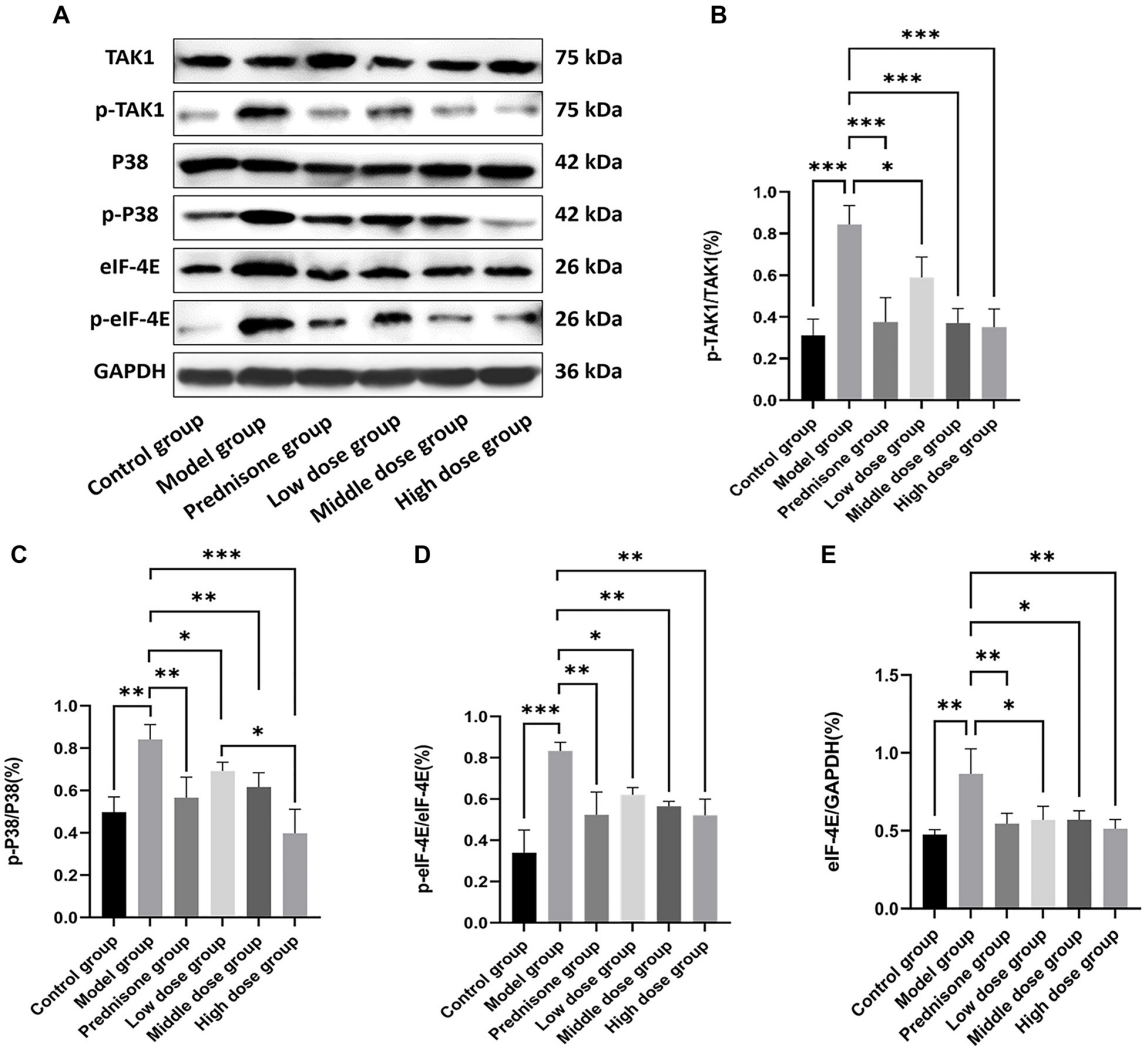
**JYBP regulates Th17 through the TAK1/P38 MAPK/eIF-4E signaling pathway.** (A) Protein levels of TAK1, p-TAK1, P38 MAPK, p-P38 MAPK, eIF-4E, and p-eIF-4E were observed by Western blot; (B) Quantitative analysis of TAK1 phosphorylation; (C) Quantitative analysis of P38 MAPK phosphorylation; (D) Quantitative analysis of eIF-4E phosphorylation; (E) Quantitative analysis of eIF-4E total protein. *n* ═ 3. *P < 0.05, **P < 0.01, ***P < 0.001. JYBP: Jianpi Yiqi Busui Prescription; elf-4E: Eukaryotic initiation factor 4E; TAK1: Transforming growth factor beta-activated kinase 1; P38 MAPK: P38 mitogen-activated protein kinase.

Based on previous research data and scientific hypotheses, this study further explores the regulatory mechanisms of JYBP on Th17 and IL-17 in the EAMG rat model. Western blot analysis was used to detect key proteins and their phosphorylation involved in the TAK1/P38 MAPK/eIF-4E signaling pathway in the spleen tissue of rats from each group. The proteins analyzed included TAK1, p-TAK1, P38, p-P38, eIF-4E, and p-eIF-4E. Representative protein bands are shown in Figure 7A, while the quantitative analysis of the grayscale values of the protein bands across the groups is presented in Figure 7B–7E. In the model group, total eIF-4E protein levels and the phosphorylation levels of TAK1, P38 MAPK, and eIF-4E in spleen tissue were significantly elevated compared to the control group. Both prednisone and JYBP effectively inhibited these expression levels and prevented overactivation of the signaling pathway, thereby reducing Th17 and IL-17 production. Overall, the inhibitory effects were more pronounced in the prednisone group, the medium-dose JYBP group, and the high-dose JYBP group.

**Figure 8.**
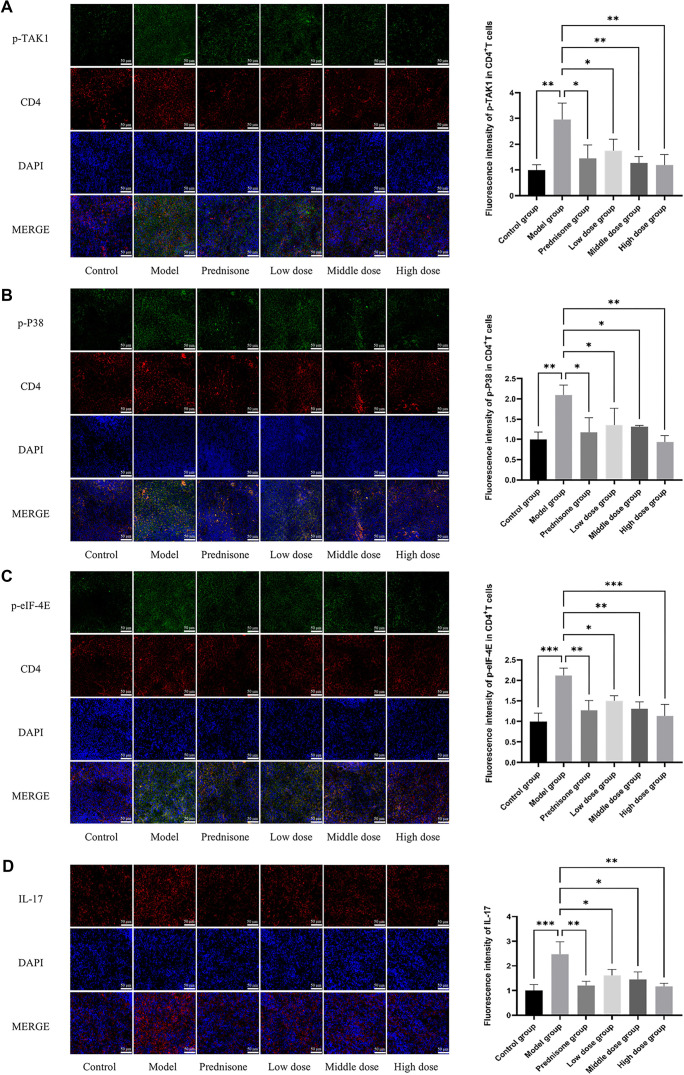
**Fluorescence intensity of IL-17 and key protein phosphorylation in the TAK1/P38 MAPK/eIF-4E signaling pathway in the spleen tissue of rats.** (A) Co-localization of CD4 and p-eIF-4E; (B) Co-localization of CD4 and p-P38 MAPK; (C) Co-localization of CD4 and p-eIF-4E; (D) IL-17. The immunofluorescence images are shown on the left, with corresponding quantitative analysis results are presented on the right. The magnification for all images is 40x, with a scale bar of 50 µm. *n* ═ 3. *P < 0.05, **P < 0.01, ***P < 0.001. elf-4E: Eukaryotic initiation factor 4E; TAK1: Transforming growth factor beta-activated kinase 1; P38 MAPK: P38 mitogen-activated protein kinase.

To further validate the mechanisms by which JYBP regulates Th17 cells through the TAK1/P38 MAPK/eIF-4E signaling pathway and to confirm the accuracy of the Western blot results, immunofluorescence staining was utilized to detect p-TAK1, p-P38, and p-eIF-4E within CD4^+^ T cells in the spleen tissue of rats. The results aligned with our hypothesis and corroborated previous research findings. The fluorescence intensity of p-TAK1, p-P38 MAPK, and p-eIF-4E in the model group was significantly higher compared to the control group. However, treatment with prednisone and JYBP led to a noticeable decrease in their fluorescence intensity (Figure 8A–8C).

Additionally, immunofluorescence was used to detect IL-17 in spleen tissue, with results similarly indicating that JYBP reduces IL-17 fluorescence intensity (Figure 8D). Western blot and immunofluorescence techniques served as complementary methods for detecting target proteins, providing mutual validation. In this study, Western blotting was employed for the quantitative analysis of protein expression levels, while immunofluorescence was used to investigate the distribution of specific proteins. The results from both methodologies were consistent, underscoring the reliability of the experimental findings.

## Discussion

Our previous network pharmacology and molecular docking study, based on HPLC analysis, indicates that the core active components of JYBP for treating MG are quercetin, epigallocatechin-3-gallate, luteolin, wogonin, kaempferol, and fisetin [25].

Quercetin has been shown to inhibit the release of inflammatory cytokines by suppressing the NF-κB and P38 MAPK signaling pathways. It also increases the CD4/CD8 ratio in thymus and spleen lymphocytes, reduces lymphocyte apoptosis, promotes immune cell proliferation, restores the Th1/Th2 and Treg/Th17 balance, and mitigates immune function damage caused by inflammatory responses [34–36].

Epigallocatechin-3-gallate regulates immune function through its anti-inflammatory and antioxidant properties. Studies have demonstrated its ability to inhibit the expression of inflammatory cytokines, such as IL-1β, IL-6, and TNF-α, prevent IL-17A-mediated CCL20 production, suppress P38 MAPK and ERK activity, reduce IL-17 expression, and influence the differentiation of naïve CD4^+^ T cells into various effector subgroups, thereby aiding in the treatment of autoimmune diseases [37–39].

Luteolin exerts anti-inflammatory and immune-regulating effects by modulating signaling pathways like P38 MAPK, JNK, and NF-κB, as well as the expression of related cytokines and kinases [40, 41].

Wogonin displays anti-inflammatory, antioxidant, and immune-regulating properties. It reduces the release of inflammatory cytokines, such as IL-17 and IL-1β, by inhibiting signaling pathways like P38 MAPK and NF-κB, and regulates Th17 cell expression [42–44].

Kaempferol helps regulate the Th17/Treg balance and IL-17 secretion by suppressing the overactivation of the JNK and P38 MAPK signaling pathways [45, 46].

Fisetin downregulates Th1/Th17 cell expression and IL-17 secretion by inhibiting the PI3K/AKT/mTOR and MAPK signaling pathways [47].

Collectively, these studies suggest that the active components of JYBP commonly exhibit anti-inflammatory, antioxidant, and immune-regulating effects in various diseases. These effects are primarily linked to their ability to regulate the differentiation of CD4^+^ T cell subgroups, such as Th17, and modulate cytokine expression, like IL-17, through pathways such as P38 MAPK. However, there is currently no research indicating whether these components exert similar effects in the treatment of MG. Addressing this question is the primary focus of our study.

This study strictly followed the guidelines for standard preclinical experiments using the EAMG animal model [27]. The model was evaluated based on several indicators, including body weight, Lennon clinical score, forelimb grip strength, and AChR-Ab concentration. To improve the accuracy, objectivity, and reliability of screening for qualified EAMG model rats, RNS of electromyography was also incorporated into the evaluation. RNS is a crucial diagnostic indicator in the clinical assessment of MG [48].

Following model establishment, MG-related symptoms in the rats appeared as expected and progressively worsened with an increasing number of immunization injections. Due to factors, such as weakened muscle strength and difficulty chewing and eating, the model rats exhibited weight loss, decreased forelimb grip strength, and higher Lennon clinical scores. Additionally, the decrement in action potential wave amplitude observed in RNS suggests a transmission impairment at the NMJ, likely due to elevated AChR-Ab concentrations [49]. These changes collectively confirm the successful establishment of the EAMG rat model.

In assessing the efficacy of JYBP in treating EAMG model rats, the recommended guidelines were followed, utilizing the Lennon clinical score, forelimb grip strength, AChR-Ab concentration, and body weight as evaluation parameters [27]. Additionally, the rotarod test was incorporated as an outcome measure to assess the fatigue resistance of the model rats [50]. The results of this study demonstrate that JYBP effectively alleviates the condition of the model rats, reduces AChR-Ab expression levels, and increases both body weight and forelimb grip strength. These findings align with results from other TCM formulations used in the treatment of MG [51, 52]. However, JYBP shows a more pronounced advantage in reducing the Lennon clinical score and enhancing fatigue resistance compared to other TCM formulations [52].

In this study, the therapeutic effects were more pronounced in the JYBP high-dose group, JYBP medium-dose group, and prednisone group, while the JYBP low-dose group exhibited relatively inferior effects, with statistically significant differences in body weight and forelimb grip strength compared to the other treatment groups. We speculate that this may be due to insufficient drug dosage. Notably, during the first two weeks of treatment, the prednisone group showed rapid and significant improvement in condition, body weight, and Lennon clinical score. However, in the later stages of treatment, some rats in the prednisone group experienced a reemergence of symptoms and slight weight loss. We speculate that these outcomes may be related to the side effects of long-term steroid use [53].

Unlike previous studies on the mechanisms of TCM formulations for MG [20, 54], this study innovatively elucidates the mechanism of action of JYBP in treating MG by inhibiting the differentiation of CD4^+^ T cells into Th17 cells. Furthermore, experimental results suggest that this process may be associated with the TAK1/P38 MAPK/eIF-4E signaling pathway.

To investigate whether the treatment of JYBP in EAMG model rats is related to the balance of CD4^+^ T cell subpopulations and Th17-related cytokines, we first used flow cytometry to detect Th17, Th1, Th2, and Treg cells in the spleen tissue of rats. The results show that JYBP can upregulate Th2 and Treg cells while reducing Th17 and Th1 cells. By regulating the differentiation of CD4^+^ T cell subpopulations, it promotes immune balance in the body, similar to the mechanisms of many other drugs used to treat MG [15, 55].

In the latter stages of the research, we focused on Th17 as the primary subject and analyzed Th17-related cytokines using ELISA and RT-qPCR techniques. The results from both methods were generally consistent, indicating that both prednisone and JYBP inhibit the expression of pro-inflammatory cytokines, such as IL-17, IL-21, IL-23, TNF-α, IL-1β, and IL-6, along with their corresponding mRNA levels. Additionally, they promote the expression of the anti-inflammatory cytokine TGF-β and its mRNA.

We found that the JYBP high-dose group exhibited a more pronounced regulatory effect on the mRNA expression levels of certain cytokines, such as IL-17, TNF-α, and TGF-β, with statistically significant differences compared to the low-dose group. Another noteworthy finding is that while the ELISA results for IL-17, IL-1β, and IL-6 in the JYBP low-dose group showed statistically significant differences compared to the model group, their corresponding mRNA expression levels did not exhibit significant differences.

We speculate that this discrepancy may be due to JYBP affecting a specific step in the process of mRNA translation into proteins, potentially involving translation factors that lead to reduced protein expression. This could explain the statistically significant differences in protein levels despite unchanged mRNA levels. After reviewing relevant literature, we believe that eIF-4E may be an important factor contributing to this phenomenon, particularly concerning IL-17 [24]. We acknowledge that the interpretation of these cytokine results should be approached with caution. Although statistically significant differences were observed between the intervention and model groups, the magnitude of change for some cytokines was not substantial. Nevertheless, studies suggest that even minor changes in these cytokines may significantly impact MG or other immune-related diseases [56–58]. Therefore, the biological significance of these changes warrants further exploration and validation, particularly in larger-scale experiments.

Based on the results of this study, it can be argued that the efficacy of JYBP in treating EAMG model rats may be related to its ability to regulate the balance of Th17, Th1, Th2, and Treg cells. The regulation of Th17 cells may, in turn, be associated with JYBP’s influence on the expression of IL-1β, IL-6, TGF-β, TNF-α, IL-21, and IL-23.

Although preliminary studies have clarified the regulatory effects of JYBP on Th17 cells and their related cytokines, further investigation is needed to elucidate its precise mechanism of action. Based on our published research and other relevant literature [23, 25], we speculate that the overexpression of TNF-α, IL-1β, IL-6, and other cytokines in EAMG model rats may lead to the phosphorylation of TAK1 in CD4^+^ T cells via the action of TRAF6, TAB1, and TAB2 [59]. Phosphorylated TAK1 is expected to further promote the phosphorylation of P38 MAPK [60], thereby enhancing Th17 differentiation and IL-17 expression by activating eIF-4E in CD4^+^ T cells during translation.

JYBP appears to reduce the expression levels of proteins involved in the TAK1/P38 MAPK/eIF-4E signaling pathway in CD4^+^ T cells of EAMG model rats, thereby inhibiting Th17 differentiation and IL-17 expression [24]. To validate this hypothesis, we employed Western blotting to detect the expression levels of proteins within the TAK1/P38 MAPK/eIF-4E pathway. The results indicated that both prednisone and JYBP significantly reduced total eIF-4E protein levels, as well as the phosphorylation levels of TAK1, P38 MAPK, and eIF-4E.

To further confirm these findings, we used immunofluorescence staining to detect p-TAK1, p-P38, and p-eIF-4E within CD4^+^ T cells in the spleen tissue of rats. The immunofluorescence results were consistent with the Western blot data, and IL-17 staining results aligned with previous IL-17 detection findings. These results support the hypothesis that JYBP exerts inhibitory effects on Th17 differentiation and IL-17 expression through the TAK1/P38 MAPK/eIF-4E signaling pathway.

The P38 MAPK signaling pathway is involved in various physiological and pathological processes, such as immunity and inflammation [61, 62], and plays a regulatory role in Th17 cells and IL-17 [63, 64]. Current experimental studies using the experimental allergic encephalomyelitis (EAE) animal model indicate that inhibiting P38 MAPK in CD4^+^ T cells of EAE rats decreases the expression of Th17 and IL-17, thereby reducing nerve damage [65]. Conversely, when P38 MAPK is specifically activated, IL-17 expression increases, and the severity of the disease worsens [66]. Further research shows that the P38 MAPK signaling pathway affects Th17 and IL-17 at both the transcriptional and translational levels. At the transcriptional level, P38 MAPK can activate ATF2/CREB, which binds to the cAMP response element (CRE) of the IL-17 gene to promote its transcription [67, 68]. At the translational level, P38 MAPK can regulate Th17 and IL-17 by affecting eEF2K or indirectly influencing eIF-4E through MNK in CD4^+^ T cells [24, 69, 70]. Additionally, variations in the total protein levels of eIF-4E also appear to influence the expression of Th17 and IL-17 [71, 72]. Furthermore, some studies suggest that the regulation of Th17 by P38 MAPK may be linked to its specific transcription factor, RORγt [73]. However, other research does not support this conclusion [74], indicating ongoing divergence in the field.

Previous studies using various animal models have clearly demonstrated that the P38 MAPK signaling pathway regulates the differentiation of Th17 cells and the expression of IL-17 in CD4^+^ T cells. However, no research has yet confirmed whether this conclusion holds true in the EAMG animal model. This study aims to fill that gap. Additionally, this study innovatively elucidates that the regulatory mechanism of JYBP on Th17 cells and IL-17 expression in EAMG model rats may be linked to the TAK1/P38 MAPK/eIF-4E signaling pathway.

Based on statistical data from all the study results, no significant differences were found between the medium- and high-dose JYBP groups and the prednisone group. This suggests that, in terms of various indicators, a sufficient dose of JYBP is as effective as prednisone. Moreover, as a TCM therapy, JYBP has the potential to avoid the side effects associated with long-term prednisone use [14]. Overall, these findings provide stronger support for the application of JYBP in the clinical treatment of MG.

However, for successful clinical translation, the safety, dose-response relationship, and potential side effects of JYBP in treating MG still require further toxicological, pharmacological, and clinical analysis. We plan to investigate these aspects in future research. While our study offers evidence that JYBP alleviates MG by inhibiting the differentiation of CD4^+^ T cells into Th17 cells through the TAK1/P38 MAPK/eIF-4E signaling pathway, several limitations should be noted. First, due to certain constraints, this study focuses solely on the mechanism by which JYBP regulates Th17 cells, lacking further exploration of its effects on other CD4^+^ T cell subtypes and B cells. Second, as JYBP is a formula composed of various traditional Chinese medicinal ingredients, its regulatory effects on Th17 cells may involve additional targets that warrant further investigation. Addressing these aspects will be a key focus in our subsequent research. Currently, we are conducting clinical studies on JYBP for MG to promote its broader application in clinical practice.

## Conclusion

In conclusion, JYBP enhances muscle strength and fatigue resistance in EAMG model rats, reduces serum AChR-Ab concentrations, alleviates MG-related symptoms, and demonstrates significant therapeutic effects. Experimental studies show that JYBP regulates the differentiation of Th17, Th1, Th2, and Treg cells in the spleen tissue of EAMG model rats, as well as the expression levels of Th17-related cytokines. Further research suggests that JYBP’s inhibitory effects on Th17 and IL-17 may be linked to its regulation of the TAK1/P38 MAPK/eIF-4E signaling pathway in CD4^+^ T cells. Based on the data collected, there appears to be no significant difference between a sufficient dose of JYBP and prednisone, indicating that JYBP holds promise as a complementary and alternative therapy for MG treatment. Ultimately, our research aims to further validate the efficacy and elucidate the mechanism of action of JYBP, supporting its development as an effective treatment for MG.

## Data Availability

Data and figures used in this study can be obtained from the corresponding author upon reasonable request.
